# Shifting to Plant-Based Protein Diets Alters Nutrient Adequacy Across Age Groups: A Dutch Dietary Modeling Study

**DOI:** 10.3390/nu18132127

**Published:** 2026-07-01

**Authors:** Jan de Vries, Cécile M. Singh-Povel, Lizette A. A. C. M. Oudhuis, Paul de Vos, Renate Akkerman

**Affiliations:** 1 Nutrition Solutions, Gorssel, The Netherlands; 2 FrieslandCampina, 3818 LE Amersfoort, The Netherlands; cecile.singh-povel@frieslandcampina.com; 3 Van Hall Larenstein University of Applied Science, 8901 BV Leeuwarden, The Netherlands; a.a.c.m.oudhuis@pl.hanze.nl; 4 Centre for Healthy Eating & Food Innovation (HEFI), Sustainable Foods and Health Group, Maastricht University—Campus Venlo, Villafloraweg 1, 5928 SZ Venlo, The Netherlands; paul.devos@maastrichtuniversity.nl

**Keywords:** animal-based proteins, diet, essential amino acids, micronutrients, plant-based proteins

## Abstract

**Background:** Shifting toward more plant-based diets is promoted for health, environmental, and ethical reasons. However, the nutritional consequences of reducing animal-based foods, particularly across age groups with specific dietary needs, remain insufficiently understood. **Methods:** In this simulation study, we used dietary intake data from 3570 participants in the Dutch National Food Consumption Survey (2019–2021) to evaluate how replacing animal-based foods with plant-based alternatives affects the intake of protein, essential amino acids (EAAs), vitamins, and minerals across different age groups. Two substitution scenarios were modeled: a nutritionally conscious and a less conscious plant-based dietary pattern. **Results:** Total protein intake decreased in both scenarios, with the strongest reductions observed in elderly individuals (71–79 years). Vitamin intake, particularly B vitamins and vitamin A, declined in most age groups, and vitamin D remained chronically low. Mineral intake also decreased, notably for calcium, iron, iodine, selenium, and zinc, especially among women and adolescents. **Conclusions:** These results underscore the importance of dietary planning and targeted fortification when promoting plant-based eating patterns. Future research should refine bioavailability estimates and evaluate the long-term health effects of such dietary transitions across life stages.

## 1. Introduction

The adoption of plant-based diets in Western society is becoming increasingly important due to its health, environmental, and ethical benefits [1,2]. Diets rich in fruits, vegetables, legumes, nuts, and whole grains are linked to lower risks of chronic diseases such as cardiovascular disease, type 2 diabetes, and certain cancers [2]. Additionally, reducing reliance on animal-based diets significantly contributes to environmental sustainability by lowering greenhouse gas emission, deforestation, and water consumption. Ethical concerns regarding animal welfare further drive the transition toward plant-based diets, ultimately supporting healthier populations and more sustainable food systems [1].

Despite these numerous advantages, plant-based diets, encompassing a wide range of dietary patterns, including flexitarian, vegetarian, and vegan diets, each with distinct macro- and micronutrient profiles, also present nutritional challenges, particularly for vulnerable populations such as the elderly and children who have increased dietary requirements [3,4,5]. One key concern involves the protein quality of plant-based diets. Compared to animal-derived proteins, plant-based products generally contain less protein and have less essential amino acids per gram protein [6]. Without careful planning, plant-based diets may not provide sufficient amounts of these vital essential amino acids, potentially leading to deficiencies [4]. These amino acids, including methionine, leucine, and lysine are critical for many different processes in the body [6,7,8]. As all different organs and tissues in the body require essential amino acids for their maintenance and growth, insufficient intake of total protein and essential amino acids can impact health, especially in the elderly, who have increased needs and lower intakes, lower (quality) protein intake may, e.g., accelerate muscle loss and cause frailty [4,9]. In children, insufficient intake of protein and essential amino acids may cause growth retardation [10,11].

In addition to protein quality, other nutrients such as vitamins and minerals also require careful consideration when transitioning to plant-based diets. Several key nutrients, including iron, calcium, vitamin B2, vitamin B12 and iodine are often found in lower quantities and are sometimes in addition less bioavailable in plant-based foods compared to animal-derived sources [12]. Iron from plant sources, for example, is predominantly in the non-haem form, which has substantially lower absorption than the haem iron found in meat [13]. Calcium absorption from plant sources can be inhibited by naturally occurring compounds such as oxalates and phytates [14]. Vitamins B2 and B12, which play essential roles in energy metabolism and neurological function, are predominantly present in animal products, making them harder to obtain from plant-based diets without fortification or supplementation [12,15,16]. Iodine, often derived from dairy and seafood, can also be insufficient in plant-based patterns unless iodized salt and fortified foods are consumed [17]. These considerations highlight the importance of mindful dietary planning to ensure adequate micronutrient intake during a shift toward plant-based eating.

One critical question is whether individuals adopting plant-based diets can still meet the recommended nutrient intakes, especially if they shift significantly away from animal-based foods. Studies show that such shift may compromise intake of key micronutrients like calcium, vitamin B12 and iron without the use of fortified foods or supplements [18]. In the Dutch context, Borkent et al. (2024) demonstrated that replacing animal-based foods with plant-based alternatives significantly reduced utilizable protein intake in older adults, especially in vegan diets, where up to 60% did not meet estimated requirements. This highlights the need for careful dietary planning to maintain nutrient adequacy in plant-based transitions [19].

Despite these insights, several gaps in the literature remain. First, there is limited understanding of how nutrient adequacy varies across different age groups within plant-based diets. Vulnerable populations, such as children and older individuals may have increased risks of nutrient deficiency due to their specific physiological and metabolic needs [20,21]. Second, existing research often assumes an idealized “nutritionally conscious” scenario, where individuals prioritize diverse food choices and include fortified products or supplements in their diet. However, not all individuals adopting plant-based diets follow such practices, resulting in varying nutrient intake scenarios.

Here we simulated observationally a transition toward a more plant-based diet to roughly estimate the impact on protein intake, essential amino acids, and other key micro- and macronutrients across different age groups, ranging from newborns to older adults. For this simulation experiment we used dietary intake data obtained from the 2019–2021 Dutch National Food Consumption Survey. The simulation provides a detailed estimate of how dietary shifts influence nutrient adequacy in populations with diverse nutritional needs. This approach enables the identification of potential nutritional deficiencies when substituting products containing animal-based proteins with products containing plant-based proteins. Our simulation experiment intends to highlight the importance of tailored strategies, such as incorporating fortified foods or supplements, to ensure optimal nutrient intake across all demographics. Through this work, we aim to offer actionable insights that support the safe and sustainable adoption of plant-based diets while meeting the nutritional requirements of varied age groups in the Dutch population.

## 2. Materials and Methods

### 2.1. Study Design

For this study, data from the Dutch National Food Consumption Survey (DNFCS 2019–2021) [22] was used, which is a periodical, cross-sectional food consumption survey conducted by the National Institute for Public Health and The Environment (RIVM) [22]. For this survey, a market research agency (Kantar) designed and recruited a survey population that represents the Dutch inhabitants based on the following sociodemographic factors: age, gender, region, degree of urbanization and education level. In waves of 4 weeks, invitations were sent to the representatives and after each wave, the composition and size of the sample were adjusted based on the response obtained in the previous waves. The overall response rate was 36.8% (3570 out of 9701 people invited). An additional non-response analysis was not performed as part of the present study. Individuals aged 1–79 were included in the DNFCS 2019–2021, and were further divided into male and female age groups of 1–3, 4–8, 9–13, 14–17, 18–30, 31–50, 51–64 and 65–79. Data from all these groups were included in our study [22] (Supplementary Table S1).

### 2.2. Dietary Intake of Protein and Essential Amino Acid Content

Food consumption data was collected via two non-consecutive 24 h dietary recalls per participant using the GloboDiet program [23]. Both recalls were performed within 2–6 weeks of each other [22]. Intake of protein and other macro- and micronutrients was calculated based on the Dutch food composition database [24,25].

A transition towards a vegan dietary pattern with plant-based protein can influence both intake of total protein quantity, as well as protein quality ((essential) amino acid composition). As the Dutch food composition database does not provide essential amino acid content of food products, we estimated the average essential amino acid content for animal protein products, plant protein products, cereal protein products and legume protein products using the Danish food consumption table [26]. To this end, we selected a variety of products from all different groups (Supplementary Table S2), which we used to estimate average amounts of essential amino acid per gram of protein for each group (Table 1). Histidine was included as well. The essentiality for histidine has been shown only for infants; however, small amounts are probably needed for adults as well. To date, the indispensability of histidine has not been documented in healthy adults [27].

### 2.3. Simulation of Dietary Scenarios

To assess the impact of a transition towards a more plant-based dietary pattern, different scenarios were created representing a nutritious conscious (scenario 1) and a nutritious unconscious scenario (scenario 2). The distinction between the nutritious conscious (scenario 1) and nutritious unconscious (scenario 2) patterns was based on differences in the typical nutritional quality of plant-based choices observed in the Dutch market. Scenario 1 reflects a more deliberate selection of nutritionally favorable substitutes, such as products with higher protein content or lower levels of processing. In contrast, scenario 2 represents a less deliberate pattern, using plant-based alternatives that are widely available but often lower in nutritional quality. These distinctions were implemented by selecting representative items within the different food group that differ in processing level and nutrient composition, thereby capturing realistic variation in consumer choices. Supplementary Table S3 provides an overview of the specific products assigned to each scenario.

For both scenarios, in the Netherlands commonly consumed animal-based product groups were selected and included in the replacement scenarios. The substitution scenarios were not designed to reflect a fully vegan or vegetarian diet. Only major animal-based product groups that substantially contribute to protein intake (meat, poultry, fish, dairy) were replaced by plant-based alternatives, while mixed dishes and products containing small amounts of animal-derived ingredients remained unchanged. As such, the scenarios represent a partial, incremental shift toward more plant-based eating patterns, rather than a complete elimination of all animal products.

Plant-based substitutes were chosen based on products that are widely available and commonly consumed within the Dutch market, and that function as direct analogs to the animal-based products they replaced (e.g., plant-based drinks for dairy, meat analogs for minced meat). The intention was to model a realistic, incremental shift toward more plant-based eating, rather than an idealized or nutritionally optimized pattern. Because plant-based alternatives differ considerably in nutrient composition, we selected representative items for the different NEVO food groups.

Replacements with other products were done in gram-for-gram substitution on a product basis. This was done to ensure a standardized and reproducible modeling framework across all replacements. Replacement by equal product weights does not necessarily represent realistic portion sizes or nutritional equivalence between animal-based and plant-based foods. However, using fixed gram weights allowed us to isolate the nutritional effects of replacing animal-derived foods while avoiding additional assumptions about consumer compensation or portion size adjustments. This modeling choice should therefore be interpreted as a controlled comparison of nutrient shifts under standardized replacement conditions, rather than as a reflection of real-world eating behavior.

In selecting plant-based substitutes for the replacement scenarios, we did not explicitly consider whether products were fortified. Replacement items were chosen based on commonly consumed products within each NEVO food group, and although some original products in participants’ diets may have been fortified, the plant-based replacement items were not systematically fortified. Therefore, potential effects of fortification on micronutrient intake were not modeled. Table 2 shows which product groups were replaced by alternatives for both scenarios. Further details of products within the product groups that were replaced can be found in Supplementary Table S3.

### 2.4. Impact of the Different Scenarios on Dietary Intake of Macro- and Micronutrient and Comparison to Intake Recommendations

To assess whether the intake of more plant-based foods in the different scenarios resulted in changes in the intake of macro- and micronutrients, including total protein, essential amino acids, vitamins, and minerals, we analyzed the average intake of these nutrients per age group for both the original dietary data and the data obtained after the replacement of animal-based foods by plant-based alternatives in scenario 1 and scenario 2. In addition, we evaluated whether the average intake of each nutrient per age group was above or below established daily intake recommendations (Supplementary Table S4). These reference values were derived from international authoritative bodies: total protein and essential amino acid requirements from the WHO/FAO/UNU 2007 report on ‘Protein and Amino Acid Requirements in Human Nutrition [28]; most vitamin and mineral recommendations from the European Food Safety Authority (EFSA) [29]; vitamin D from the Dutch Health Council [30]; and iodine from WHO/UNICEF/ICCIDD guidelines [31]. Mean nutrient intakes were compared with the corresponding dietary reference values (e.g., Recommended Dietary Allowances (RDA), adequate intakes (AI), or equivalent recommended intake levels; Supplementary Table S4). Because these reference values represent recommended intakes rather than estimated average requirements (EARs), the comparisons provide only an approximate indication of potential inadequacy at the population level.

No correction was applied to protein or essential amino acid recommendations for differences in protein quality. Although meals with a PDCAAS or DIAAS < 1 indicate that one or more essential amino acids fall below the scoring pattern, we did not adjust intake values for the reduced utilizable protein fraction that may result from this limiting amino acid. As some recommendations are dependent on body weight, we used the average bodyweight per age groups to calculate the corresponding recommendation levels (Supplementary Tables S1 and S4). Whenever the group AI was below the RDA, this was interpreted as ‘not meeting’ the recommendation.

## 3. Results

### 3.1. Total Protein Intake Decreases When Switching to More Plant-Based Dietary Scenarios

**Figure 1:**
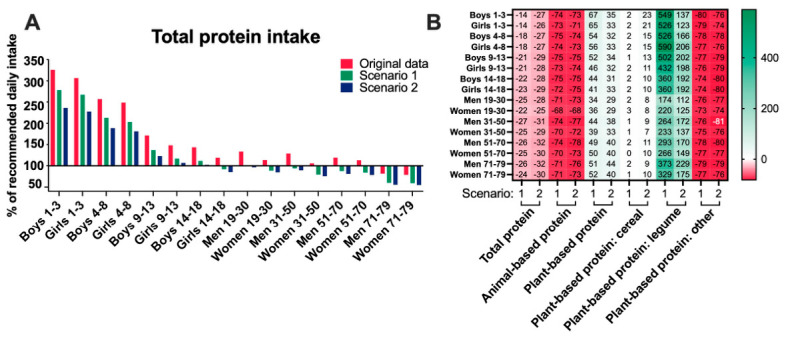
(**A**) Percentage of estimated total protein intake across different age groups based on the original dietary data and two dietary scenarios, compared to the World Health Organization (WHO) recommendation levels for age groups under 71 years and ESPEN recommended intake levels for age groups above 71 years and (**B**) changes in the percentage of protein intake for the two dietary scenarios. Average protein intake is shown for the original dataset as well as for scenario 1 (nutritionally conscious) and scenario 2 (nutritionally unconscious) dietary shifts. Data is presented as average per group.

In total, data from 3570 participants were included in this simulation study (Van Rossum et al., 2023) [22]. First, we compared the total protein intake (calculated as estimated means) across different age groups in the original dataset to the recommended daily intake levels established by the WHO (Supplementary Table S4). For the age group 71–79 we compared these intake levels to the ESPEN recommendations. Our analysis revealed that protein intake exceeded the recommended levels for all age groups, except for men and women aged 71–79, who, on average, consumed only 81.6% and 78.8% of their daily recommended intake, respectively (Figure 1A, red bars) (Supplementary Table S4).

In scenario 1, the nutritionally conscious scenario, 24,405 out of 204,532 dietary records were replaced. This dietary shift resulted in a reduction in average total protein intake across all age groups. Among females, all age groups older than 18 years no longer met the recommended daily protein intake. Similarly, among males, average total protein intake fell below the recommended daily levels for individuals aged 31–50 years and older (Figure 1A, green bars). In scenario 2, the nutritionally unconscious scenario, 27,230 out of 204,532 dietary records were modified. This resulted in a further reduction in average total protein intake across all age groups. Among females, all age groups older than 14–18 years no longer met the recommended daily protein intake. Similarly, among males, protein intake dropped below the recommended daily levels for all age groups older than 19–30 years (Figure 1A, blue bars).

To further evaluate the impact of transitioning to a diet with a higher proportion of plant-based proteins on overall protein intake, we estimated the percentage of change in not only total protein intake but also in the intake of animal-based and plant-based proteins for the two scenarios. Additionally, plant-based protein intake was further categorized into protein derived from cereal products, protein from legume-based products and protein from other products to provide a more detailed analysis (Figure 1B; Supplementary Table S2). For all age groups total protein intake decreased. More specifically, intake of animal-based proteins reduced from −68% for women 19–30 in both scenarios to up to −78% for men 51–70 in scenario 2. Logically, intake of plant-based proteins increased for all age groups in both scenarios, with a stronger effect observed for scenario 1. Percentages of intake of plant-based protein from cereal products only slightly increased in both scenarios for all age groups, while intake of plant-based proteins from legume products increased to a much higher extend.

### 3.2. Intake of Essential Amino Acids Decreases in Both Scenarios

In addition to total protein intake, we also evaluated how the intake of essential amino acids (EAAs) was affected by the transition toward a more plant-based diet in our two dietary scenarios. We estimated changes in EAA intake for both the original data and the two dietary scenarios and compared them to recommended intake levels (Supplementary Table S4).

**Figure 2:**
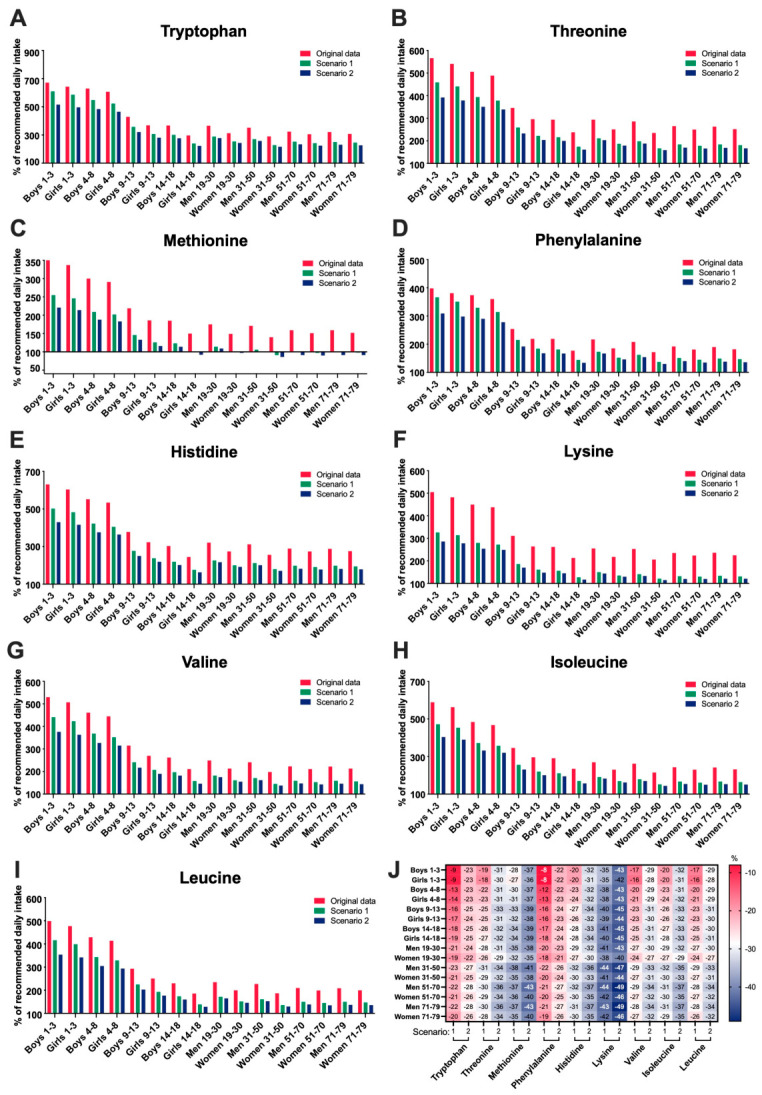
Estimated percentage of essential amino acid (EAA) intake across different age groups based on the original dietary data and two dietary scenarios, compared to the WHO recommended intake levels (**A**–**I**) as well as percentage of change in intake for both scenarios compared to the original intake (**J**). EAA intake is shown for the original dataset as well as for scenario 1 (nutritionally conscious) and scenario 2 (nutritionally unconscious) dietary shifts. Data is presented as average per group.

In the original dataset, EAA intake exceeded the recommended levels for all age groups (Figure 2A–I, red bars). However, when shifting to more plant-based dietary patterns in both scenario 1 (nutritionally conscious) and scenario 2 (nutritionally unconscious), the intake of EAAs generally decreased across all age groups (Figure 2, green and blue bars). Despite this reduction, overall EAA intake remained above the recommended levels for most age groups. An exception was observed for methionine + cysteine (Figure 2C), where intake fell below the recommended levels in older age groups in both plant-based scenarios.

### 3.3. Vitamin Intake Changes Similarly in Both Dietary Scenarios When Shifting to Plant-Based Diets

The intake of vitamins also changes with dietary changes towards a more plant-based diet. In the original dataset, most vitamins were consumed at levels close to or higher than the recommended intake, while in the dietary scenarios we generally observed (minor) reductions in intake levels.

**Figure 3:**
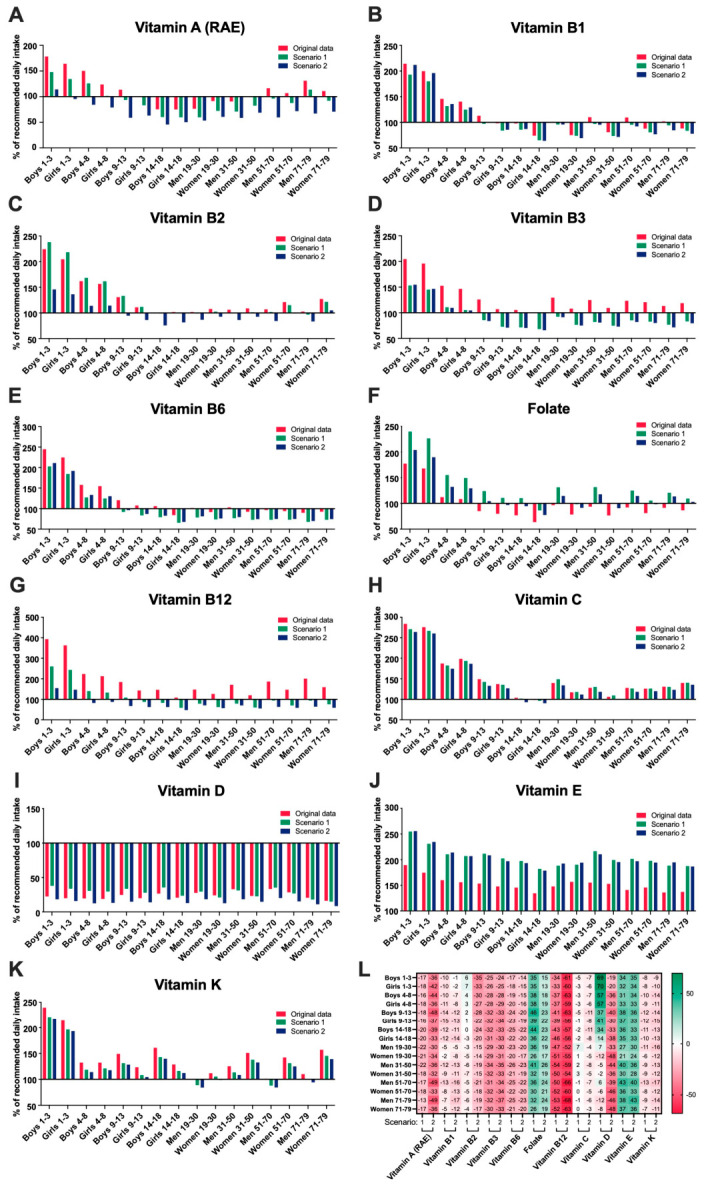
Percentages of vitamin intake across different age groups based on the original dietary data and two dietary scenarios, compared to the recommended intake levels (**A**–**K**) as well as percentage of change in intake for both scenarios compared to the original intake (**L**). Vitamin intake is presented for the original dataset as well as for scenario 1 (nutritionally conscious) and scenario 2 (nutritionally unconscious) dietary shifts. Data is presented as average per group.

For vitamins A, B1, B2, B3, B6, and B12, mean intake generally decreased among individuals older than nine years in both dietary scenarios, resulting in levels falling below the recommended intake (Figure 3). Conversely, folate intake, which was below recommended levels in the original dataset for individuals above nine years of age, slightly increased in both plant-based scenarios. As a result, folate intake met recommended levels again in male groups but remained insufficient for females (Figure 3F).

Vitamin D intake was already below recommended levels in the original dataset for all age groups, and this did not change following the dietary modifications (Figure 3I). In contrast, vitamin E intake was above the recommended levels in the original dataset and further increased in both plant-based scenarios, reflecting the higher consumption of plant-based foods rich in this nutrient (Figure 3J).

These findings highlight that while a shift to a more plant-based diet may slightly reduce the intake of certain vitamins, it can also improve the intake of others. However, specific attention may be needed to ensure adequate intake of vitamins A, B1, B2, B3, B6, B12, and D in predominantly plant-based diets.

### 3.4. The Intake of Minerals Is Affected in Both Scenarios

**Figure 4:**
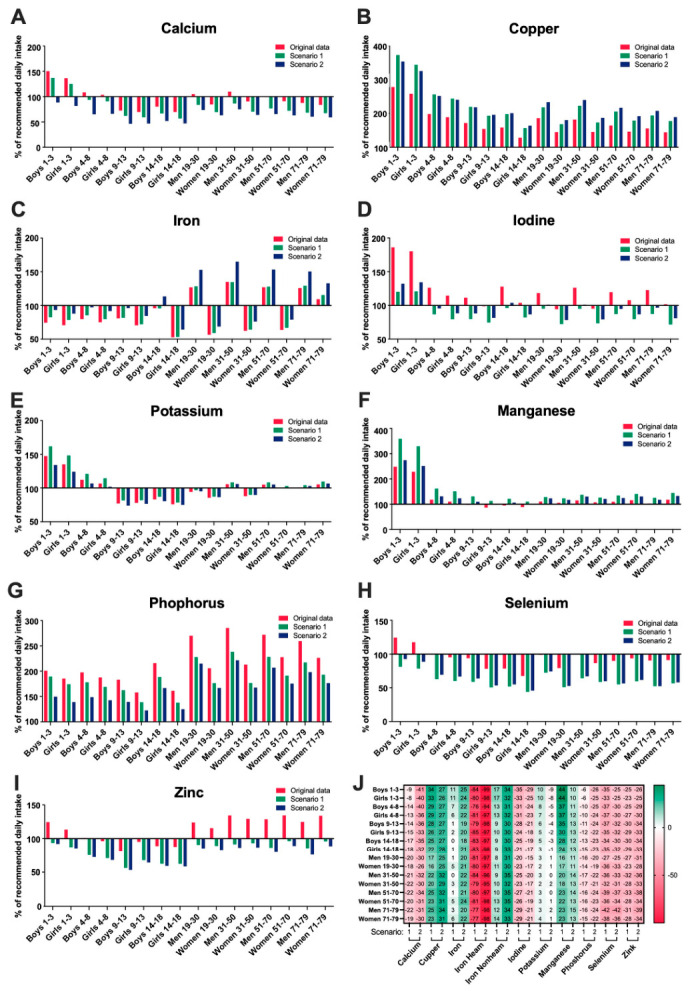
Percentages of mineral intake across different age groups based on the original dietary data and two dietary scenarios, compared to the recommended intake levels (**A**–**I**) as well as percentage of change in intake for both scenarios compared to the original intake (**J**). Mineral intake is presented for the original dataset as well as for scenario 1 (nutritionally conscious) and scenario 2 (nutritionally unconscious) dietary shifts. Data is presented as average per group.

The intake of several key minerals was below recommended levels in the original dataset, indicating potential inadequacies in mineral consumption across various age groups (Figure 4). The transition to more plant-based diets further influenced mineral intake, with varying effects depending on the specific nutrient.

Calcium intake was below recommendation levels in the original dataset for all age groups above 4 years old, except for men aged 19–30 years and men aged 31–50, and decreased in both scenarios, falling below the recommended levels across all age groups, including men aged 19–30 and men aged 31–50 (Figure 4A). Copper intake, on the other hand, which was already above the recommended levels in the original dataset, increased further in both dietary scenarios (Figure 4B). For iron, intake was already insufficient in children up to 13 years old and in women aged 14–51 years in the original dataset. While iron intake slightly increased in both scenarios, it remained below the recommended levels for these groups (Figure 4C). Similarly, iodine intake, which was generally around recommendation levels in the original dataset, decreased in both plant-based scenarios. This reduction was particularly pronounced in women aged 9 years and older, resulting in intake levels falling below recommendations (Figure 4D).

Potassium intake was below recommendation levels for individuals aged 9–30 years and women aged 31–51 years in the original dataset, with minimal changes observed in both dietary scenarios (Figure 4E). Manganese and phosphorus intake remained around recommendation levels in all conditions (Figure 4F,G). For selenium, intake was already below recommended levels in age groups older than 4 years and decreased further in both plant-based scenarios (Figure 4H). Likewise, zinc intake, which was insufficient for individuals aged 4–18 years in the original dataset, dropped below recommended levels for all age groups in both scenarios (Figure 4G).

Figure 4J presents the percentage changes in mineral intake for both dietary scenarios relative to the original dataset. In addition to the minerals described before, we examined changes in haem and non-haem iron intake, even though specific recommendations for these iron subtypes do not exist. The distinction between haem and non-haem iron is relevant, as plant-based diets are typically associated with lower bioavailability of iron [14]. In our scenarios, the intake of haem iron decreased while the intake of non-haem iron slightly increased (Figure 4J).

## 4. Discussion

This simulation study assessed the potential nutritional implications of a shift toward more plant-based diets across different age groups using dietary intake data from a representative sample of the Dutch population. By simulating two dietary scenarios, one nutritionally conscious and one nutritionally unconscious scenario, we estimated changes in protein intake, essential amino acid (EAA) sufficiency, and the adequacy of vitamin and mineral intake. Our findings showed that protein and EAA intake were particularly sensitive to the type of substitution, with elderly individuals (71–79 years) most at risk of inadequacy in the nutritionally unconscious scenario. In contrast, changes in vitamin and mineral intake were observed across both scenarios and were largely independent of substitution quality. Intakes of several micronutrients, including B vitamins, calcium, iron, iodine, selenium, and zinc, frequently fell below recommended levels, while vitamin D intake remained consistently low across all groups. These results highlight that protein adequacy depends strongly on the quality of substitutions, whereas micronutrient adequacy might present a broader challenge in plant-based dietary transitions.

At baseline, total protein intake exceeded WHO recommendations in most age groups; however, elderly participants (71–79 years) did not meet the higher intake levels recommended by ESPGHAN (1.2 g/kg body weight). These higher intake levels are considered more appropriate for maintaining muscle mass and function in aging populations. When modeling the substitution of animal-based foods with plant-based alternatives, total protein intake decreased in both dietary scenarios, with the largest reductions observed in older adults. The nutritionally unconscious scenario had the strongest impact, leading to protein adequacy being compromised in several additional age groups. For EAA intake, most remained above recommendations across both scenarios; however, methionine fell below recommended levels in some older age groups, while lysine remained close to but slightly above the requirement. These findings suggest that methionine is particularly critical in plant-rich diets, with lysine also representing a borderline limiting amino acid, especially in elderly populations. Our findings align with recent Dutch research by Borkent et al. (2024) [19], who reported that in vegan dietary scenarios, up to 60% of older adults had a protein intake below the estimated average requirement for utilizable protein. Furthermore, our analysis identified methionine and lysine as respectively inadequate and borderline sufficient in elderly groups. This aligns with the paper by Wanders and colleagues (2025) which indicated cysteine, methionine, lysine and leucine as limiting AA for several reference patterns [32].

In our study, total intake of protein and amino acids were reported as absolute intake values. However, not all ingested protein and amino acids are equally available to the body. It is known that plant-based proteins generally have lower digestibility than animal-based proteins [33]. In addition, if a meal has a DIAAS below one, a substantial part of the amino acids are oxidized rather than being used for protein synthesis [34]. Since requirement values are based on high-quality, highly digestible proteins, comparisons with absolute intake may overestimate true adequacy. Utilizable protein, which corrects for digestibility and amino acid composition, provides a more realistic measure. SHARP-modeling studies by Wanders et al. have shown that in diets with ~60% plant protein, utilizable protein intake is 10–30% lower than absolute intake, and a similar effect is likely present in our scenarios [32]. Consequently, our estimates of protein and amino acid adequacy are conservative. Furthermore, for sulfur amino acids we compared intake of methionine alone against the combined requirement for methionine + cysteine, which may have led to an overestimation of inadequacy for this group.

Vitamin intake was notably affected by plant-based transitions. In the original dietary data, intake of several vitamins, including A, B1, B6, B12, D, and folate, already fell below recommended levels in various age groups. The modeled scenarios showed further declines, especially in B vitamins such as B2 and B12, which are largely derived from animal products and are not easily replaced by plant-based sources [12]. This pattern has been confirmed in previous research, including studies by Craig (2009) [35], Niklewicz et al. (2024) [18], and more recently Lawrence et al. (2025) [36], who modeled the replacement of cow’s milk with plant-based drinks in the Australian population and observed substantial reductions in riboflavin (vitamin B2), vitamin B12, protein, and iodine intake [36]. These nutrients are particularly critical for neurological function, energy metabolism, and developmental health [37,38]. Our results also confirm that vitamin D intake remains structurally inadequate across all age groups, in line with broader European trends related to limited sun exposure and low intake of fortified foods [39].

Mineral intake was similarly compromised. Even in the original data, calcium intake was below recommended levels for most individuals above 4 years old, with further reductions seen in the plant-based scenarios. Iron intake was already insufficient among children and women of reproductive age and, despite slight increases in the plant-based scenarios, remains a concern due to the lower bioavailability of non-haem iron and the presence of absorption inhibitors like phytates [14]. Zinc and iodine followed similar patterns, with intake in female adolescents and adults dropping below recommended levels after dietary shifts. Selenium, already insufficient in the original diet, decreased even more. These findings are supported by multiple studies showing that plant-based diets, unless carefully managed or fortified, are often inadequate in providing these minerals [40].

Our study benefits from several strengths, including the use of a large and representative dataset and individualized dietary substitution modeling. However, there are still opportunities for further refinement. The modeled scenarios are based on two 24 h dietary recalls from the DNFCS. Although this method is standard in dietary surveillance, it is subject to recall bias and substantial day-to-day variability, meaning that it does not fully capture habitual intake patterns. As no additional correction for these sources of variability was applied, some uncertainty in the exact intake estimates, both in the baseline and the modeled scenarios, should be acknowledged. In addition, we did not weight the dataset, though the broad population coverage helps mitigate this limitation.

A further methodological consideration is the use of gram-for-gram substitutions when replacing animal-based foods with plant-based alternatives. Equal product weights do not necessarily correspond to realistic portion sizes, consumer behavior, or nutritional equivalence; for example, 200 g of meat and 200 g of tofu differ substantially in energy density and protein content. We selected this standardized approach to ensure comparability across all substitutions and to isolate the nutrient composition effects of replacing animal-based foods, without introducing additional assumptions about portion size adjustments or compensatory eating patterns. As such, the modeled outcomes of this study reflect controlled substitution effects rather than real-world consumption patterns, and the magnitude of nutrient changes should be interpreted with this limitation in mind.

Finally, it is important to note that several of the nutrient patterns observed in our scenarios, such as lower potassium and iron intakes or the specific shifts in amino-acid adequacy, reflect the structure-preserving substitution approach rather than the nutritional characteristics of intentionally composed plant-based diets. Because the model replaces only animal-based items while maintaining the rest of the dietary pattern unchanged, it does not incorporate the legumes, vegetables, fruits, and whole grains that typically raise potassium and total iron intake in real-world plant-based eating. The scenarios were deliberately designed to identify potential nutritional points of attention that may arise when consumers make simple, product-for-product substitutions, rather than to represent an optimized or comprehensive plant-based diet. As such, the nutrient shortfalls observed here should be interpreted as indicators of aspects that may require attention during a transition, rather than predictions of inadequacy in well-planned plant-based eating patterns. Additionally, we did not apply more advanced modeling techniques such as those used in SHARP [32], which incorporate cultural feasibility and food choice behaviors. Nevertheless, our two-scenario approach provides a transparent and interpretable framework, which may be especially useful for stakeholders such as policymakers, nutrition professionals, and food producers.

Observational studies of individuals following vegetarian and vegan diets provide additional context for interpreting the findings of the current study. Large cohort and cross-sectional analyses from Europe, South/East Asia, and North America have consistently shown that well-planned plant-based diets can meet or exceed recommended intakes for protein and most essential amino acids, largely due to higher consumption of legumes, whole grains, soy products, nuts, and seeds [41,42]. At the same time, these studies also report recurrent shortfalls in specific micronutrients, including vitamin B12, vitamin D, calcium, iodine, zinc, and in some cases iron, particularly among individuals who do not use fortified foods or supplements [41,42]. Such patterns have for example been documented in the EPIC-Oxford cohort [43]. These real-world data align with several of the nutrient vulnerabilities identified in our modeling framework, while also illustrating that actual plant-based eaters often compensate for reduced animal-source foods through increased consumption of nutrient-dense plant foods, fortification, or supplementation. Thus, although our scenarios do not represent optimized plant-based diets, the nutrient shortfalls observed here correspond with well-documented nutritional points of attention during dietary transitions toward more plant-based eating.

In summary, our findings reveal nutritional vulnerabilities that may arise during the transition to more plant-based diets, particularly in more restrictive patterns such as veganism. Importantly, several inadequacies were already present at baseline, including insufficient intakes of vitamin D, calcium, iron, iodine, selenium, and zinc. Shifting to more plant-based protein further increased the prevalence of shortfalls, even when nutritionally preferred alternatives were chosen (scenario 1). This suggests that substitution alone may not always be sufficient to ensure adequacy, and that additional strategies, such as greater dietary variety, careful food combinations, and the use of fortified products or supplements, are needed to meet recommendations. It is important to note that the plant-based alternatives used in the current modeling study were based on commonly consumed products in the Dutch market and did not systematically include fortified options. Therefore, the suggestion that targeted fortification may support nutrient adequacy should be interpreted as a potential policy implication rather than a direct conclusion, and future modeling could further explore fortified and non-fortified substitution scenarios. By including a wide range of age groups in this study, from infants to older adults, our study also shows that the nutritional impact of dietary change is not uniform: older adults and adolescents are particularly at risk of insufficiency.

## 5. Conclusions

These insights provide a strong rationale for age-specific dietary guidance. For the food industry, this highlights opportunities to develop fortified or nutrient-enhanced plant-based alternatives that deliver high-quality protein and essential micronutrients. For policymakers, our results emphasize the importance of dietary recommendations and educational strategies tailored to different life stages, alongside consideration of regulatory measures on fortification. It is important to emphasize that the scenarios modeled in this study are indicative rather than predictive and do not capture all behavioral or physiological complexities. Moreover, our analysis is based on intake data and not on biomarker-based nutritional status; therefore, monitoring nutrient status in the Dutch population will be crucial to understand the true health implications of dietary transitions. Future research should also incorporate protein quality, nutrient bioavailability, and realistic consumption patterns to identify dietary solutions that support both health and sustainability.

## Figures and Tables

**Table 1:** Estimated average amounts of protein and essential amino acids per product group.

	Protein per 100 g Product	EAA (mg/100 g Product)										
		Tryp	Thr	Meth	Cyst	Phe	Tyr	His	Lys	Val	Isoleu	Leu
Animal protein products	16.2	201.2	686.8	450.8	119.2	735.1	680.5	508.7	1431.2	982.4	850.2	1385.8
Plant protein products	7.6	108.5	279.4	140.3	143.3	408.4	239.8	197.6	341.2	424.5	334.7	601.2
Cereal protein products	9.6	117.2	313.7	174.0	188.9	493.1	258.9	231.3	330.8	523.1	404.4	741.0
Legume protein products	11.5	177.7	316.5	150.6	136.3	437.3	284.1	221.5	439.4	445.5	362.1	635.3
		**EAA (mg/g protein)**										
		**Tryp**	**Thr**	**Meth**	**Cyst**	**Phe**	**Tyr**	**His**	**Lys**	**Val**	**Isoleu**	**Leu**
Animal protein		12.4	42.4	27.8	7.4	45.4	42.0	31.4	88.3	60.6	52.5	85.5
Plant protein		14.3	36.8	18.5	18.9	53.7	31.6	26.0	44.9	55.9	44.0	79.1
Cereal protein products		12.2	32.8	18.2	19.7	51.5	27.0	24.1	34.5	54.6	42.2	77.4
Legume protein products		15.4	27.4	13.1	11.8	37.9	24.6	19.2	38.1	38.6	31.4	55.0

**Table 2:** Replacement scenarios for dietary patterns with more plant-based protein products shown per food group.

Category	Product Groups (NEVO Code List: Supplementary Table S3)	Scenario 1 ‘Nutritious Conscious’: Replacement by (NEVO Code)	Scenario 2 ‘Nutritious Unconscious’: Replacement by (NEVO Code)
Dairy products and substitutes	Milk, milk beverages and fermented milk beverages	Drink Soja (3180)	Oat drink (5463)
	Cheeses (including spread cheeses)	Hummus naturel (3207)	Chocolate sprinkles, milk (1962)
Meat, meat products and substitutes	Processed meat (meats for bread)	Vegetarian luncheon meat (5478)	Tomato tapenade (5100)
	Meat, meat products and substitutes (warm meals)	Vegetarian ham (2544)	Vegetable burger (5515)
	Chicken and other poultry products (warm meals)	Fava bean (962)	Vegetable burger (5515)
Fish, shellfish and amphibians	Fish, shellfish and amphibians (warm meals)	Grean pea (972)	Vegetable burger (5515)

## Data Availability

The original contributions presented in this study are included in the article/Supplementary Materials. Further inquiries can be directed to the corresponding author.

## Supplementary Materials

The following supporting information can be downloaded at: https://www.mdpi.com/article/10.3390/nu18132127/s1, Table S1: Demographics of participants included in this study; Table S2: Protein products used to calculate average EAA contents for the different food groups; Table S3: Product groups that were replaced by alternatives in the substitution models 1 and 2; Table S4: Overview of intake recommendations per age group used in our model.

## Abbreviations

The following abbreviations are used in this manuscript:

AI	Average Intake
DNFCS	Dutch National Food Consumption Survey
EAA	Essential Amino Acid
EAR	Estimated Average Requirement
ESPEN	European Society for Clinical Nutrition and Metabolism
RDA	Recommended Daily Allowance
WHO	World Health Organization

## References

[1] Moughan P.J. (2021). Population protein intakes and food sustainability indices: The metrics matter. Glob. Food Secur..

[2] Xiao X., Zou P.-R., Hu F., Zhu W., Wei Z.-J. (2023). Updates on plant-based protein products as an alternative to animal protein: Technology, properties, and their health benefits. Molecules.

[3] Kiely M.E. (2021). Risks and benefits of vegan and vegetarian diets in children. Proc. Nutr. Soc..

[4] Neufingerl N., Eilander A. (2023). Nutrient intake and status in children and adolescents consuming plant-based diets compared to meat-eaters: A systematic review. Nutrients.

[5] Fouillet H., Huneau J., Perraud E., Dussiot A., Wang J., Kesse-Guyot E., Mariotti F. (2025). Plant to animal protein ratio in the diet of the elderly: Potential for increase and impacts on nutrient adequacy and long-term health—A diet optimization study. Am. J. Clin. Nutr..

[6] Gorissen S.H.M., Crombag J.J.R., Senden J.M.G., Waterval W.A.H., Bierau J., Verdijk L.B., van Loon L.J.C. (2018). Protein content and amino acid composition of commercially available plant-based protein isolates. Amino Acids.

[7] Pinckaers P.J.M., Trommelen J., Snijders T., van Loon L.J.C. (2021). The anabolic response to plant-based protein ingestion. Sports Med..

[8] Ling Z., Jiang Y., Ru J., Lu J., Ding B., Wu J. (2023). Amino acid metabolism in health and disease. Signal Transduct. Target Ther..

[9] Aggarwal R., Bains K. (2022). Protein, lysine and vitamin D: Critical role in muscle and bone health. Crit. Rev. Food Sci. Nutr..

[10] Semba R.D., Shardell M., Sakr Ashour F.A., Moaddel R., Trehan I., Maleta K.M., Ordiz M.I., Kraemer K., Khadeer M.A., Ferrucci L. (2016). Child stunting is associated with low circulating essential amino acids. eBioMedicine.

[11] Budzulak J., Majewska K.A., Kędzia A. (2022). Malnutrition as the cause of growth retardation among children in developed countries. Ann. Agric. Environ. Med..

[12] Chungchunlam S.M.S., Moughan P.J. (2024). Comparative bioavailability of vitamins in human foods sourced from animals and plants. Crit. Rev. Food Sci. Nutr..

[13] Johnston B.C., Zeraatkar D., Han M.A., Vernooij R.W.M., Valli C., El Dib R., Marshall C., Stover P.J., Fairweather-Taitt S., Wójcik G. (2019). Unprocessed red meat and processed meat consumption: Dietary guideline recommendations from the nutritional recommendations (NutriRECS) consortium. Ann. Intern. Med..

[14] Amalraj A., Rius A. (2015). Bioavailability of calcium and its absorption inhibitors in raw and cooked green leafy vegetables commonly consumed in India—An in vitro study. Food Chem..

[15] Calderón-Ospina C.A., Nava-Mesa M.O. (2019). B vitamins in the nervous system: Current knowledge on the biochemical modes of action and synergies of thiamine, pyridoxine, and cobalamin. CNS Neurosci. Ther..

[16] Obeid R., Heil S.G., Verhoeven M.M.A., van den Heuvel E.G.H.M., de Groot L.C.P.G.M., Eussen S.J.P.M. (2019). Vitamin B12 intake from animal foods, biomarkers and health aspects. Front. Nutr..

[17] Nicol K., Nugent A.P., Woodside J.V., Hart K.H., Bath S.C. (2024). The impact of replacing milk with plant-based alternatives on iodine intake: A dietary modelling study. Eur. J. Nutr..

[18] Niklewicz A., Hannibal L., Warren M., Ahmadi K.R. (2024). A systematic review and meta-analysis of functional vitamin B12 status among adult vegans. Nutr. Bull..

[19] Borkent J.W., Grootswagers P., Linschooten J., Roodenburg A.J.C., Ocké M., van der Schueren M.A.E. (2025). A Vegan dietary pattern is associated with high prevalence of inadequate protein intake in older adults; a simulation study. J. Nutr. Health Aging.

[20] Awuchi C.G., Igwe V.S., Amagwula I.O. (2020). Nutritional diseases and nutrient toxicities: A systematic review of the diets and nutrition for prevention and treatment. Int. J. Adv. Acad. Res..

[21] Kiani A.K., Dhuli K., Donato D., Aquilanti B., Velluti V., Matera G., Iaconelli A., Connelly S.T., Bellinato F., Gisondi P. (2022). Main nutritional deficiencies. J. Prev. Med. Hyg..

[22] Van Rossum C.T.M., Sanderman-Nawijn E.L., Brants H.A.M., Dinnissen C.S., Jansen-Van der Vliet M., Beukers M.H., Ocké M.C. The Diet of the Dutch. Results of the Dutch National Food Consumption Survey 2019–2021 on Food Consumption and Evaluation with Dietary Guidelines [Internet]. RIVM 2023. https://www.rivm.nl/publicaties/diet-of-dutch-results-of-dutch-national-food-consumption-survey-2019-2021-on-food.

[23] Slimani N., Casagrande C., Nicolas G., Freisling H., Huybrechts I., Ocké M.C., Niekerk E.M., van Rossum C., Bellemans M., De Maeyer M. (2011). on behalf of the ERCOVAL Consortium. The standardized computerized 24-h dietary recall method EPIC-Soft adapted for pan-European dietary monitoring. Eur. J. Clin. Nutr..

[24] RIVM (2021). NEVO-Online 2021 (Version 2021/7.0). National Institute for Public Health and the Environment (RIVM). https://www.rivm.nl/en/dutch-food-composition-database?utm_source=chatgpt.com.

[25] Jansen-van der Vliet M., Westenbrink S., Niekerk E.M., Roos A.M., Van den Bogaard-van Oosterhout C.H.M. (2021). NEVO-Online 2021; Background Information.

[26] National Food Institute, Technical University of Denmark, Danish Veterinary and Food Administration (2023). The Danish Food Composition Database (FRIDA), Version 5.0: Documentation. https://frida.fooddata.dk.

[27] Gheller M.E., Vermeylen F., Handzlik M.K., Gheller B.J., Bender E., Metallo C., Aydemir T.B., Smriga M., Thalacker-Mercer A.E. (2020). Tolerance to graded dosages of histidine supplementation in healthy adults. Am. J. Clin. Nutr..

[28] World Health Organization, Food and Agriculture Organization of the United Nations, United Nations University (2007). Protein and Amino Acid Requirements in Human Nutrition: Report of a Joint WHO/FAO/UNU Expert Consultation.

[29] European Food Safety Authority (EFSA) (2024). Dietary Reference Values. https://www.efsa.europa.eu/en/topics/topic/dietary-reference-values.

[30] Health Council of the Netherlands (2012). Evaluation of the Dietary Reference Values for Vitamin D.

[31] World Health Organization, United Nations Children’s Fund, International Council for the Control of Iodine Deficiency Disorders (2007). Assessment of Iodine Deficiency Disorders and Monitoring Their Elimination: A Guide for Programme Managers.

[32] Wanders A.J., Heerschop S.N., Biesbroek S., Dötsch-Klerk M. (2025). Replacing animal meat with plant-based meat alternatives: The impact of protein quality on protein adequacy in the Dutch diet. Curr. Dev. Nutr..

[33] Hertzler S.R., Lieblein-Boff J.C., Weiler M., Allgeier C. (2020). Plant proteins: Assessing their nutritional quality and effects on health and physical function. Nutrients.

[34] Wolfe R.R., Rutherfurd S.M., Kim I., Moughan P.J. (2016). Protein quality as determined by the digestible indispensable amino acid score: Evaluation of factors underlying the calculation. Nutr. Rev..

[35] Craig W.J. (2009). Health effects of vegan diets. Am. J. Clin. Nutr..

[36] Lawrence A.S., Russo-Batterham D., Doyle K., Tescari E. (2025). Time to consider more than just calcium? The impact on protein, riboflavin, vitamin B12 and iodine intake of replacing cow’s milk with plant-based milk-like drinks—An Australian usual intake dietary modelling study. Eur. J. Nutr..

[37] Nawaz A., Khattak N.N., Khan M.S., Nangyal H., Sabri S., Shakir M. (2020). Deficiency of vitamin B_12_ and its relation with neurological disorders: A critical review. J. Basic Appl. Zool..

[38] Fuzi S.F.A., Loh S.P. (2022). Iodine: A critical micronutrient in brain development. Role of Micronutrients in Brain Health, Nutritional Neurosciences.

[39] Benedik E. (2022). Sources of vitamin D for humans. Int. J. Vitam. Nutr. Res..

[40] Pellinen T., Päivärinta E., Isotalo J., Lehtovirta M., Itkonen S.T., Korkalo L., Erkkola M., Pajari A. (2022). Replacing dietary animal-source protein with plant-source protein changes dietary intake and status of vitamins and minerals in healthy adults: A 12-week randomized controlled trial. Eur. J. Nutr..

[41] Neufingerl N., Eilander A. (2021). Nutrient intake and status in adults consuming plant-based diets compared to meat-eaters: A systemic review. Nutrients.

[42] Bakaloudi D.R., Halloran A., Rippin H.L., Oikonomidou A.C., Dardavsis T.I., Williams J., Wickramasinghe K., Breda J., Chourdakis M. (2021). Intake and adequacy of the vegan diet. A systemic review of the evidence. Clin. Nutr..

[43] Key J.T., Papier K., Tong T.Y.N. (2021). Plant-based diets and long-term health: Findings from the EPIC-Oxford study. Prot. Nutr. Soc..

