# Supplementary material for: Shifting to Plant-Based Protein Diets Alters Nutrient Adequacy Across Age Groups: A Dutch Dietary Modeling Study
Source: Nutrients. 2026 Jul 1;18(13):2127. doi: 10.3390/nu18132127 (PMC13363323; doi:10.3390/nu18132127)
Supplement: Supplementary file 1 [file nutrients-18-02127-s001.zip › nutrients-4322170-supplementary.pdf]

Supplementary materials

Shifting to plant-based protein diets alters nutrient adequacy across age-groups: A Dutch dietary modelling study

Supplementary Table 1. Demographics of participants included in this study

Age group	Average length ± stdev (cm)	Average bodyweight ± stdev (kg)	Average BMI ± stdev
Boys 1-3	91,4 (8,5)	14,0 (2,5)	16,7 (1,4)
Girls 1-3	91,2 (9,1)	13,6 (2,7)	16,3 (1,5)
Boys 4-8	121,8 (11,2)	24,2 (5,6)	16,1 (1,7)
Girls 4-8	120,7 (11,1)	23,6 (5,6)	16,0 (1,8)
Boys 9-13	153,9 (11,7)	44,6 (11,9)	18,6 (3,0)
Girls 9-13	152,5 (11,3)	44,7 (11,7)	19,0 (3,2)
Boys 14-18	178,2 (8,0)	66,9 (12,8)	21,0 (3,3)
Girls 14-18	168,2 (6,8)	61,6 (10,4)	21,8 (3,3)
Men 19-30	183,6 (7,2)	80,2 (13,3)	23,8 (3,8)
Women 19-30	169,9 (6,6)	70,0 (13,7)	24,3 (4,8)
Men 31-50	183,1 (7,1)	88,8 (16,3)	26,5 (4,4)
Women 31-50	169,6 (7,3)	78,6 (16,5)	27,3 (5,5)
Men 51-70	180,5 (7,6)	90,1 (15,1)	27,7 (4,6)
Women 51-70	166,6 (7,5)	77,8 (15,8)	28,1 (5,6)
Men 71-79	178,1 (7,1)	87,8 (12,5)	27,1 (4,5)
Women 71-79	164,8 (6,3)	76,2 (14,0)	26,8 (5,3)

Supplementary Table 2. Protein products used to calculate average EAA contents for the different food groups

Category	FoodGroup ID Frida	Food Group Name
Animal protein products from Frida database (EAA)	4	Meat and meat products
	5	Fish, aquatic animals, and their products
	8	Poultry
	12	Soft sour milk cheese
	13	Firm rennet cheese
	14	Soft rennet cheese
	15	Semi-firm rennet cheese
	16	Whey cheese
	17	Processed cheese
	57	Other meat and fresh meat products
	58	Beef
	59	Veal
	60	Pork
	61	Sheep and lamb
	72	Somewhay oily fish
	73	Oily fish
	77	Mollusks and their products
	79	Fish products
	81	Chicken
	82	Turkey
	84	Duck and goose
	87	Fresh eggs
	90	Egg products
	183	Other aquatic animal products
Plant protein products from Frida database (EAA)	28	Grain and groats
	30	Flour and bran
	31	Bread
	34	Breakfast products
	35	Pasta

	39	Root and tuber vegetables
	41	Fruit-Vegetables
	43	Mushrooms
	46	Canned vegetable products
	53	Nuts
	169	Plant-based products
	171	Fresh legumes
	172	Frozen legumes
	174	Canned legumes
	175	Other legume products
	177	High-fat seeds
	178	Nut products
	188	Other vegetable products
	189	High-fat seeds products
	191	Prepared legumes
Cereal protein products from Frida database (EAA)	28	Grain and groats
	30	Flour and bran
	31	Bread
	34	Breakfast products
	35	Pasta
Legume protein products from Frida database (EAA)	53	Nuts
	169	Plant-based products
	171	Fresh legumes
	172	Frozen legumes
	174	Canned legumes
	175	Other legume products
	177	High-fat seeds
	178	Nut products
	189	High-fat seeds products
	191	Prepared legumes

Supplementary Table 3: Product groups that were replaced by alternatives in the substitution models 1 and 2.

Category	Product groups	Scenario 1			Scenario 2		
		Nevo code	Product name (in Dutch)	Product name (in English)	Nevo code	Product name (in Dutch)	Product name (in English)
Dairy products and substitutes	Milk, milk beverages and fermented milk beverages	270	Melk gewone (koe)	Regular milk (cow)	270	Melk rauwe	Regular milk (cow)
		272	Melk chocolade- gewone	Regular chocolate milk	271	Koffiemelk	Milk coffee
		273	Melk chocolade- gewone	Regular chocolate milk	271	Koffieroom	Cream coffee
		279	Melk gewone (koe)	Regular milk (cow)	272	Melk chocolade- gewone	Regular chocolate milk
		286	Melk gewone (koe)	Regular milk (cow)	279	Melk gewone (koe)	Regular milk (cow)
		289	Karnemelk gewone	Regular buttermilk	279	Melk, n.s.	Regular milk not specified
		294	Melk gewone (koe)	Regular milk (cow)	279	Melk geen koemelk	Milk (not cow)
		295	Melkpoeder	Milkpowder	279	Melk lactosevrij	Milk (lactose free)
		296	Melkpoeder	Milkpowder	280	Koffiemelk	Milk coffee
		297	Melk moeder-	Mothermilk	280	Koffiemelk/room	Milk/cream coffee
		479	Karnemelkdrank	Buttermilk drink	285	Koffiemelk	Milk coffee
		657	Yoghurt drink-	Yogurt drink	285	Koffiemelk/room	Milk/cream coffee
		862	Milkshake	Milkshake	286	Melk gewone (koe)	Regular milk (cow)
		1464	Melk chocolade- gewone	Regular chocolate milk	286	Melk geen koemelk	Milk (not cow)
		1681	Melkpoeder	Milkpowder	286	Melk, n.s.	Milk (not specified)
		1719	Melk verrijkte (vit./min. e.d.)	Fortified milk (vitamins/minerals)	292	Koffiemelk	Milk coffee
		1813	Minidrink zuivel op yoghurtbasis	Mini yogurt-based dairy drinks	292	Koffiemelk/room vloeibaar, n.s.	Milk/cream coffee
		1832	Drinkontbijt, n.s.	Breakfast drink, not specified	294	Melk gewone (koe)	Regular milk (cow)
		1834	Yoghurt drink-	Yogurt-based drink	294	Melk gewone (koe)	Milk (not cow)
		1970	Melk chocolade- gewone	Regular chocolate drink	294	Melkdrank Milk&Fruit/Fruitmelk en soortgelijke	Milkdrink fruit etc
		2240	Melk geen koemelk	Milk (non-cow origin)	294	Melk, n.s.	Milk (not specified)
		2254	Yoghurt drink-	Yogurt-based drink	1464	Melk chocolade- gewone	Regular chocolate milk
		2255	Yoghurt drink-	Yogurt-based drink	1719	Melk verrijkte (vit./min. e.d.)	Fortified milk (vitamins/minerals)
		2256	Drinkontbijt, n.s.	Breakfast drink, not specified	2053	Melkdrank Milk&Fruit/Fruitmelk en soortgelijke	Milkdrink fruit etc
		2257	Drinkontbijt op yoghurtbasis	Yogurt-based breakfast drink	2240	Melk geen koemelk	Milk (not cow)
		2258	Minidrink zuivel op yoghurtbasis	Mini yogurt-based dairy drinks	2240	Melk, n.s.	Regular milk not specified
		2265	Minidrink zuivel op yoghurtbasis	Mini yogurt-based dairy drinks	2495	Melk chocolade- gewone	Regular chocolate milk
		2308	Minidrink zuivel op yoghurtbasis	Mini yogurt-based dairy drinks	2500	Melk chocolade- gewone	Regular chocolate milk
		2495	Melk chocolade- gewone	Regular chocolate milk	2822	Yoghurt gewone (koe) met smaakje	Yoghurt (cow), with taste
		2496	Melkdrank geen chocolade/anijs	Milk drink (not chocolate/anise)	2822	Yoghurt biogarde	Yoghurt biograde
		2497	Milkshake	Milkshake	2822	Yoghurtsnack	Yoghurt snack
		2500	Melk chocolade- gewone	Regular chocolate milk	3004	Melk chocolade- gewone	Regular milk (cow)
		2502	Yoghurt drink-	Yogurt-based drink	3234	Melkdrank Milk&Fruit/Fruitmelk en soortgelijke	Milkdrink fruit etc
		2597	Minidrink zuivel op yoghurtbasis	Mini yogurt-based dairy drinks	5245	Melk chocolade- gewone	Regular chocolate milk
		2689	Minidrink zuivel op yoghurtbasis	Mini yogurt-based dairy drinks			
		2725	Melkdrank geen chocolade/anijs	Milk drink (not chocolate/anise)			
		2760	Melk chocolade- uit automaat	Chocolate milk from vending machine			
		2921	Yoghurt drink-	Yogurt-based drink			
		2936	Melk verrijkte (vit./min. e.d.)	Fortified milk (vitamins/minerals)			
		2941	Drinkontbijt op yoghurtbasis	Yogurt-based breakfast drink			
		3004	Melk chocolade- gewone	Regular chocolate milk			
		3015	Melkpoeder	Milkpowder			
		3114	Melkpoeder	Milkpowder			
		3201	Ayran (ongeveer 60% yoghurt)	Ayran (approx. 60% yogurt)			
		3234	Melkdrank geen chocolade/anijs	Milk drink (not chocolate/anise)			
		3240	Melk lactose-arm kant-en-klaar	Ready-to-drink lactose-free milk			
		3259	Minidrink zuivel op yoghurtbasis	Mini yogurt-based dairy drinks			
		3316	Minidrink zuivel op yoghurtbasis	Mini yogurt-based dairy drinks			
		3317	Minidrink zuivel op yoghurtbasis	Mini yogurt-based dairy drinks			
		3364	Yoghurt drink-	Yogurt-based drink			
		3365	Yoghurt drink-	Yogurt-based drink			
		3430	Melkpoeder	Milkpowder			
		3443	Yoghurt drink-	Yogurt-based drink			
	Cheeses (including spread cheeses)	304	Kaas Zwitserse strooi-	Swiss grated cheese	304	Kaas Zwitserse strooi-	Swiss grated cheese
		511	Kaas Edammer	Edam cheese	511	Kaas Edammer	Edam cheese
		513	Kaas, n.s.	Cheese, not specified	513	Kaas, n.s.	Cheese, not specified
		514	Kaas, n.s.	Cheese, not specified	514	Kaas, n.s.	Cheese, not specified
		515	Kaas smeer-	Speadable cheese	515	Kaas smeer-	Speadable cheese
		516	Kaas smeer-	Speadable cheese	516	Kaas smeer-	Speadable cheese
		517	Kaas smeer-	Speadable cheese	517	Kaas smeer-	Speadable cheese
		556	Kaas camembert	Camembert cheese	556	Kaas camembert	Camembert cheese
		593	Kaas brie	Brie cheese	593	Kaas brie	Brie cheese
		654	Kaas huttenkase	Cheese, huttenkase	654	Kaas huttenkase	Cheese, huttenkase
		714	Kaas blauwschimmel-	Blue cheese	714	Kaas blauwschimmel-	Blue cheese
		716	Kaas Goudse en soortgelijke belegen	Gouda and similar semi-mature cheese	716	Kaas Goudse en soortgelijke belegen	Gouda and similar semi-mature cheese
		718	Kaas Parmezaanse	Parmesan cheese	718	Kaas Parmezaanse	Parmesan cheese
		719	Kaas room- zachte	Soft cream cheese	719	Kaas room- zachte	Soft cream cheese
		722	Kaas gaten-	Hole cheese (Swiss-style)	722	Kaas gaten-	Hole cheese (Swiss-style)
		724	Kaas gaten-	Hole cheese (Swiss-style)	724	Kaas gaten-	Hole cheese (Swiss-style)
		725	Kaas cheddar-	Cheddar cheese	725	Kaas cheddar-	Cheddar cheese

		726	Kaas blauwschimmel-	Blue cheese	726	Kaas blauwschimmel-	Blue cheese
		728	Kaas room- zachte	Soft cream cheese	728	Kaas room- zachte	Soft cream cheese
		804	Kaas feta en soortgelijke	Feta and similar cheese	804	Kaas feta en soortgelijke	Feta and similar cheese
		882	Kaas Goudse en soortgelijke belegen	Gouda and similar semi-mature cheese	882	Kaas Goudse en soortgelijke belegen	Gouda and similar semi-mature cheese
		883	Kaas Goudse en soortgelijke oude	Gouda and similar aged cheese	883	Kaas Goudse en soortgelijke oude	Gouda and similar aged cheese
		1104	Kaas rook-	Smoked cheese	1104	Kaas rook-	Smoked cheese
		1109	Kaas smeer-	Speadable cheese	1109	Kaas smeer-	Speadable cheese
		1112	Kaas boeren- rauwmelkse	Farmhouse/raw milk cheese	1112	Kaas boeren- rauwmelkse	Farmhouse/raw milk cheese
		1302	Kaas room- zachte	Soft cream cheese	1302	Kaas room- zachte	Soft cream cheese
		1382	Kaas boeren- rauwmelkse	Farmhouse/raw milk cheese	1382	Kaas boeren- rauwmelkse	Farmhouse/raw milk cheese
		1487	Kaas brie	Brie cheese	1487	Kaas brie	Brie cheese
		1489	Kaas room- zachte	Soft cream cheese	1489	Kaas room- zachte	Soft cream cheese
		1650	Kaas geiten- zachte	Soft goat cheese	1650	Kaas geiten- zachte	Soft goat cheese
		1723	Kaas Goudse en soortgelijke jong belegen	Gouda and similar young mature cheese	1723	Kaas Goudse en soortgelijke jong belegen	Gouda and similar young mature cheese
		1724	Kaas Goudse en soortgelijke jonge	Gouda and similar young cheese	1724	Kaas Goudse en soortgelijke jonge	Gouda and similar young cheese
		1725	Kaas Goudse en soortgelijke jonge	Gouda and similar young cheese	1725	Kaas Goudse en soortgelijke jonge	Gouda and similar young cheese
		1726	Kaas Goudse en soortgelijke belegen	Gouda and similar semi-mature cheese	1726	Kaas Goudse en soortgelijke belegen	Gouda and similar semi-mature cheese
		1809	Kaas Goudse en soortgelijke belegen	Gouda and similar semi-mature cheese	1809	Kaas Goudse en soortgelijke belegen	Gouda and similar semi-mature cheese
		1939	Kaas blauwschimmel-	Blue cheese	1939	Kaas blauwschimmel-	Blue cheese
		1955	Kaas Mozzarella	Mozzarella cheese	1955	Kaas Mozzarella	Mozzarella cheese
		2516	Kaas smeer-	Speadable cheese	2516	Kaas smeer-	Speadable cheese
		2517	Kaas smeer-	Speadable cheese	2517	Kaas smeer-	Speadable cheese
		2518	Kaas geiten- harde	Hard goat cheese	2518	Kaas geiten- harde	Hard goat cheese
		2678	Kaas zuivelspread	Dairy spread cheese	2678	Kaas zuivelspread	Dairy spread cheese
		2679	Kaas room- zachte	Soft cream cheese	2679	Kaas room- zachte	Soft cream cheese
		2756	Kaas Goudse en soortgelijke jonge	Gouda and similar young cheese	2756	Kaas Goudse en soortgelijke jonge	Gouda and similar young cheese
		2757	Kaas Goudse en soortgelijke jong belegen	Gouda and similar young mature cheese	2757	Kaas Goudse en soortgelijke jong belegen	Gouda and similar young mature cheese
		2758	Kaas Goudse en soortgelijke belegen	Gouda and similar semi-mature cheese	2758	Kaas Goudse en soortgelijke belegen	Gouda and similar semi-mature cheese
		2759	Kaas Goudse en soortgelijke oude	Gouda and similar aged cheese	2759	Kaas Goudse en soortgelijke oude	Gouda and similar aged cheese
		2925	Kaas Mascarpone	Mascarpone cheese	2925	Kaas Mascarpone	Mascarpone cheese
		2995	Kaas smeer-	Speadable cheese	2995	Kaas smeer-	Speadable cheese
		3044	Kaas Turks	Cheese, Turkish	3044	Kaas Turks	Cheese, Turkish
		3045	Kaas schapen-	Sheep's cheese	3045	Kaas schapen-	Sheep's cheese
		3155	Kaas Goudse en soortgelijke jonge	Gouda and similar young cheese	3155	Kaas Goudse en soortgelijke jonge	Gouda and similar young cheese
		3163	Kaas Goudse en soortgelijke jong belegen	Gouda and similar young mature cheese	3163	Kaas Goudse en soortgelijke jong belegen	Gouda and similar young mature cheese
		3164	Kaas Goudse en soortgelijke belegen	Gouda and similar semi-mature cheese	3164	Kaas Goudse en soortgelijke belegen	Gouda and similar semi-mature cheese
		3165	Kaas Goudse en soortgelijke oude	Gouda and similar aged cheese	3165	Kaas Goudse en soortgelijke oude	Gouda and similar aged cheese
		3166	Kaasproduct plantaardige vetten	Cheeseproduct plant-based fats	3166	Kaasproduct plantaardige vetten	Cheeseproduct plant-based fats
		3356	Kaas room- zachte	Soft cream cheese	3356	Kaas room- zachte	Soft cream cheese
		3368	Kaas verse	Fresh cheese	3368	Kaas verse	Fresh cheese
		3370	Kaas feta en soortgelijke	Feta and similar cheese	3370	Kaas feta en soortgelijke	Feta and similar cheese
		3377	Kaas Ricotta	Ricotta cheese	3377	Kaas Ricotta	Ricotta cheese
Meat, meat products and substitutes	Processed meat (meats for bread)	319	Corned beef	Corned beef	319	Corned beef	Corned beef
		328	Ham rauwe	Raw ham	328	Ham rauwe	Raw ham
		333	Lever gekookte als broodbeleg	Cooked liver (as sandwich meat)	333	Lever gekookte als broodbeleg	Cooked liver (as sandwich meat)
		334	Lever gekookte als broodbeleg	Cooked liver (as sandwich meat)	334	Lever gekookte als broodbeleg	Cooked liver (as sandwich meat)
		335	Leverpastei	Liver pâté	335	Leverpastei	Liver pâté
		336	Smac luncheon meat en soortgelijke	Luncheon meat and similar products (e.g., Smac)	336	Smac luncheon meat en soortgelijke	Luncheon meat and similar products (e.g., Smac)
		338	Pekelvlees	Salted meat (cured beef)	338	Pekelvlees	Salted meat (cured beef)
		340	Rookvlees	Smoked beef	340	Rookvlees	Smoked beef
		566	Worst knak-	Frankfurter sausage	567	Worst boterham-	Sandwich sausage
		567	Worst boterham-	Sandwich sausage	638	Cervelaat	Cervelat sausage
		568	Worst bloed-	Blood sausage	639	Spek ontbijt-	Breakfast bacon
		638	Cervelaat	Cervelat sausage	640	Worst lever, n.s.	Liver sausage, not specified
		639	Spek ontbijt-	Breakfast bacon	641	Bacon	Bacon
		640	Worst lever, n.s.	Liver sausage, not specified	642	Pate andere	Other påte
		641	Bacon	Bacon	643	Casselerrib	Smoked pork loin (Casselerrib)
		642	Pate andere	Other påte	782	Worst thee-	Tea sausage
		643	Casselerrib	Smoked pork loin (Casselerrib)	783	Worst paling-	Eel sausage
		782	Worst thee-	Tea sausage	784	Ham gekookte	Cooked ham
		783	Worst paling-	Eel sausage	785	Ham schouder-	Shoulder ham
		784	Ham gekookte	Cooked ham	810	Filet americain	Raw beef spread (Filet americain)
		785	Ham schouder-	Shoulder ham	1152	Salami	Salami
		810	Filet americain	Raw beef spread (Filet americain)	1155	Vleeswaar als broodbeleg, gebraden/gegrild, n.s.	Grilled/roasted sandwich meat, not specified
		1152	Salami	Salami	1162	Worst gekookte (gelderse)	Cooked sausage (Gelderland style)
		1155	Vleeswaar als broodbeleg, gebraden/gegrild, n.s.	Grilled/roasted sandwich meat, not specified	1211	Vleeswaren, laag vet	Meat for bread, low fat
		1162	Worst gekookte (gelderse)	Cooked sausage (Gelderland style)	1239	Worst smeerworst	Spreadable sausage
		1238	Worst Hausmacher	Hausmacher sausage	1367	Worst droge sucuk Turks	Dried sausage, Turkish
		1239	Worst smeerworst	Spreadable sausage	1368	Salam Turkse droge worst	Turkish dry salami
		1368	Salam Turkse droge worst	Turkish dry salami	1771	Leverkaas	Liver cheese
		1544	Rollade als broodbeleg	Pork roll (for sandwich)	1772	Fricandeau als broodbeleg	Veal roast (Fricandeau) for sandwich
		1545	Rosbief als broodbeleg	Roast beef (for sandwich)	1773	Vleeswaar als broodbeleg, n.s.	Sandwich meat, not specified
		1572	Vleeswaar als broodbeleg, gebraden/gegrild, n.s.	Grilled/roasted sandwich meat, not specified	1774	Worst tongen-	Tongue sausage
		1601	Gehakt gebraden/gegrild als broodbeleg	Cooked/grilled minced meat (for sandwich)	1775	Spek ontbijt-	Breakfast bacon
		1641	Rollade als broodbeleg	Pork roll (for sandwich)	1776	Ham gegrilde	Grilled ham
		1675	Worst chipolata	Chipolata sausage	1777	Ham been- als broodbeleg	Bone-in ham (for sandwich)
		1771	Leverkaas	Liver cheese	1907	Vleeswaar als broodbeleg, n.s.	Sandwich meat, not specified
		1772	Fricandeau als broodbeleg	Veal roast (Fricandeau) for sandwich	1909	Worst, gekookt, n.s.	Cooked sausage, not specified
		1773	Vleeswaar als broodbeleg, n.s.	Sandwich meat, not specified	2304	Salami	Salami
		1774	Worst tongen-	Tongue sausage	2306	Smac luncheon meat en soortgelijke	Luncheon meat and similar products (e.g., Smac)
		1776	Ham gegrilde	Grilled ham	2654	Kipfilet als broodbeleg	Chicken fillet (for sandwich)
		1777	Ham been- als broodbeleg	Bone-in ham (for sandwich)	2768	Worst ossen-	Raw beef sausage

		1907	Vleeswaar als broodbeleg, n.s.	Sandwich meat, not specified	2836	Worst grill- met kaas	Grilled sausage with cheese
		1909	Worst, gekookt, n.s.	Cooked sausage, not specified	2898	Rookvlees	Smoked beef
		1910	Vleeswaar als broodbeleg, n.s.	Sandwich meat, not specified	2996	Worst chorizo-	Chorizo sausage
		2304	Salami	Salami	2997	Worst grill-	Grilled sausage
		2306	Smac luncheon meat en soortgelijke	Luncheon meat and similar products (e.g., Smac)	3001	Kalkoenfilet als broodbeleg	Turkey fillet (for sandwich)
		2381	Pate andere	Other pâté	3002	Spek katen-	Smoked lean bacon
		2654	Kipfilet als broodbeleg	Chicken fillet (for sandwich)	3203	Ham, n.s.	Ham, not specified
		2836	Worst grill- met kaas	Grilled sausage with cheese	3223	Worst grill-	Grilled sausage
		2883	Rookvlees	Smoked beef	5094	Rollade als broodbeleg	Prok roll (for sandwich)
		2898	Rookvlees	Smoked beef	5165	Worst, gedroogd, n.s.	Dried sausage, not specified
		2996	Worst chorizo-	Chorizo sausage			
		2997	Worst grill-	Grilled sausage			
		3001	Kalkoenfilet als broodbeleg	Turkey fillet (for sandwich)			
		3002	Spek katen-	Smoked lean bacon			
		3003	Worst knak-	Frankfurter sausage			
		3203	Ham, n.s.	Ham, not specified			
		3223	Worst grill-	Grilled sausage			
		5094	Rollade als broodbeleg	Prok roll (for sandwich)			
		5165	Worst, gedroogd, n.s.	Dried sausage, not specified			
	Meat, meat products and substitutes (warm meals)	95	Paard biefstuk	Horse steak	95	Paard biefstuk	Horse steak
		106	Eend filet	Duck fillet	106	Eend filet	Duck fillet
		108	Kip borst	Chicken breast	107	Haas	Hare
		109	Konijn tam, n.s.	Domestic rabbit, not specified	108	Kip borst	Chicken breast
		319	Corned beef	Corned beef	109	Konijn tam, n.s.	Domestic rabbit, not specified
		322	Frikandel	Frikandel (Dutch minced meat snack)	324	Worst rook-	Smoked sausage
		324	Worst rook-	Smoked sausage	330	Kalkoen rollade	Turkey roast
		330	Kalkoen rollade	Turkey roast	339	Ree wild	Deer (wild)
		336	Smac luncheon meat en soortgelijke	Luncheon meat and similar products (e.g., Smac)	343	Spek reepjes/blokjes vet	Fatty bacon strips/cubes
		343	Spek reepjes/blokjes vet	Fatty bacon strips/cubes	566	Worst knak- blik/glas	Frankfurter sausages - canned/jarred
		638	Cervelaat	Cervelat sausage	568	Worst bloed-	Blood sausage
		643	Casselerrib	Smoked pork loin (Casselerrib)	908	Gehaktbal	Meatball
		908	Gehaktbal	Meatball	1305	Kip reepjes	Chicken strips
		1305	Kip reepjes	Chicken strips	1306	Worst braad-	Pan-frying sausage
		1306	Worst braad-	Pan-frying sausage	1390	Rund gehakt	Ground beef
		1306	Kip soep-	Chicken (for soup)	1400	Rund biefstuk	Beef steak
		1400	Rund biefstuk	Beef steak	1401	Rund ossenhaas	Beef tenderloin (filet mignon)
		1401	Rund ossenhaas	Beef tenderloin (filet mignon)	1402	Rund entrecote	Beef entrecote (boneless rib steak)
		1402	Rund entrecote	Beef entrecote (boneless rib steak)	1404	Rund lap bak-/bief-	Beef steak/braising cut
		1404	Rund lap bak-/bief-	Beef steak/braising cut	1405	Rund gehakt	Ground beef
		1405	Rund gehakt	Ground beef	1406	Rund klapstuk	Beef flank (klapstuk – fatty flank cut)
		1406	Rund klapstuk	Beef flank (klapstuk – fatty flank cut)	1407	Lever runder	Beef liver
		1408	Rund poulet	Beef for stew (poulet)	1408	Rund poulet	Beef for stew (poulet)
		1409	Rund rollade	Beef roast	1409	Rund rollade	Beef roast
		1410	Rund rosbief	Roast beef	1410	Rund rosbief	Roast beef
		1411	Rund schenkel	Beef shank	1411	Rund schenkel	Beef shank
		1412	Rund lap rib-	Beef rib cut	1412	Rund lap rib-	Beef rib cut
		1413	Rund lap doorregen	Marbled beef cut	1413	Rund lap doorregen	Marbled beef cut
		1414	Rund lap sukade-	Beef chuck/succade cut	1414	Rund lap sukade-	Beef chuck/succade cut
		1415	Rund tartaar	Beef tartare	1415	Rund tartaar	Beef tartare
		1417	Worst braad-	Pan-frying sausage	1416	Rundertong	Beef tongue
		1418	Varken lap filet-	Pork loin cut	1417	Worst braad-	Pan-frying sausage
		1419	Varken fricandeau	Pork roast (fricandeau)	1418	Varken lap filet-	Pork loin cut
		1420	Varken lap ham-	Pork ham cut	1419	Varken fricandeau	Pork roast (fricandeau)
		1421	Varken gehakt	Ground pork	1420	Varken lap ham-	Pork ham cut
		1422	Varken haas-	Pork tenderloin	1421	Varken gehakt	Ground pork
		1423	Varken hamschijf	Pork ham shank	1422	Varken haas-	Pork tenderloin
		1424	Varken karbonade haas-	Park loin chop	1423	Varken hamschijf	Pork ham shank
		1425	Varken karbonade schouder-	Pork shoulder chop	1424	Varken karbonade haas-	Park loin chop
		1427	Varken krabbetjes	Pork ribs	1425	Varken karbonade schouder-	Pork shoulder chop
		1429	Varken oester	Pork oyster cut (small, tender cut near the backbone)	1426	Lever varkens- rauw	Pork liver
		1430	Varken lap schouder-	Pork shoulder cut	1427	Varken krabbetjes	Pork ribs
		1431	Varken slavink	Pork meat roll wrapped in bacon (Slavink)	1429	Varken oester	Pork oyster cut (small, tender cut near the backbone)
		1432	Spek reepjes/blokjes, n.s.	Bacon strips/cubes, not specified	1430	Varken lap schouder-	Pork shoulder cut
		1434	Gehakt half-om-half	Mixed ground meat (Half beef, half pork)	1431	Varken slavink	Pork meat roll wrapped in bacon (Slavink)
		1435	Hamburger	Hamburger	1432	Spek reepjes/blokjes, n.s.	Bacon strips/cubes, not specified
		1436	Kalf entrecote	Veal entrecote	1434	Gehakt half-om-half	Mixed ground meat (Half beef, half pork)
		1437	Kalf blinde vink	Veal meat roll (Blinde vink)	1435	Hamburger	Hamburger
		1443	Lam bout	Lamb leg	1437	Kalf blinde vink	Veal meat roll (Blinde vink)
		1444	Worst braad-	Pan-frying sausage	1444	Worst braad-	Pan-frying sausage
		1445	Lam kotelet	Lamb chob	1445	Lam kotelet	Lamb chob
		1447	Lam schouder-	Lamb shoulder	1446	Lamszadel	Lamb saddle
		1498	Salade kip-kerrie	Chicken salade kerrie	1544	Runderrollade	Beef roulade
		1568	Gehaktbal	Meatball	1545	Runderrosbief	Roast beef
		1601	Gehaktbal	Meatball	1568	Gehaktbal	Meatball
		1634	Kip filet	Chicken fillet	1601	Gehaktbal	Meatball
		1641	Kiprollade bereid	Chicken roast	1634	Kip filet	Chicken fillet
		1642	Kip rollade	Chicken roast	1641	Kiprollade	Chicken roulade
		1643	Kip nuggets	Chicken nuggets	1642	Kip rollade	Chicken roast
		1663	Rund carpaccio	Beef carpaccio	1663	Rund carpaccio	Beef carpaccio
		1668	Varken lap, n.s.	Pork cut, not specified	1668	Varken lap, n.s.	Pork cut, not specified
		1673	Kalf, n.s.	Veal, not specified	1673	Kalf, n.s.	Veal, not specified
		1675	Lam, n.s.	Lamb, not specified	1675	Lam, n.s.	Lamb, not specified
		1788	Varken karbonade rib-	Pork rib chop	1788	Varken karbonade rib-	Pork rib chop
		1790	Varken schnitzel	Pork schnitzel	1790	Varken schnitzel	Pork schnitzel
		1936	Kalkoen filet	Turkey fillet	1936	Kalkoen filet	Turkey fillet
		2058	Schaap kotelet	Mutton chop	2057	Lamsvlees	Lamb meat
		2090	Kip drumstick	Chicken drumstick	2089	Struisvogel biefstuk	Ostrich steak
		2300	Worst rook-	Smoked sausage	2090	Kip drumstick	Chicken drumstick
		2301	Worst rook-	Smoked sausage	2300	Worst rook-	Smoked sausage
		2306	Smac luncheon meat en soortgelijke	Luncheon meat and similar products (e.g., Smac)	2301	Worst rook-	Smoked sausage
		2333	Gehakt, n.s.	Ground meat, not specified	2302	Worst knak- magere blik/glas	Frankfurter sausages - canned/jarred
		2334	Gehaktbal	Meatball	2313	Rundvlees gezouten gekookt Surinaams	Salted beef (Surinamese)
		2336	Rund, n.s.	Beef, not specified	2333	Gehakt, n.s.	Ground meat, not specified

		2337	Gehaktbal	Meatball	2334	Gehaktbal	Meatball
		2338	Rund stoofvlees, n.s.	Stewing beef, not specified	2335	Vlees	Meat
		2339	Rund vink	Beef meat roll (vink)	2336	Rund, n.s.	Beef, not specified
		2340	Varken schnitzel	Pork schnitzel	2337	Gehaktbal	Meatball
		2341	Kip filet	Chicken fillet	2338	Rund stoofvlees, n.s.	Stewing beef, not specified
		2342	Kip borst	Chicken breast	2339	Rund vink	Beef meat roll (vink)
		2344	Cordon bleu (gewone)	Cordon blue (regular)	2340	Varken schnitzel	Pork schnitzel
		2362	Frikandel	Frikandel (Dutch minced meat snack)	2341	Kip filet	Chicken fillet
		2363	Boomstammetje (vlees)	Boomstammetje (minced meat roll with sauce)	2342	Kip borst	Chicken breast
		2364	Kipschnitzel (gepaneerd met paneermeel)	Breaded chicken schnitzel	2344	Cordon bleu (gewone)	Cordon blue (regular)
		2365	Cordon bleu (gewone)	Cordon blue (regular)	2363	Boomstammetje (vlees)	Boomstammetje (minced meat roll with sauce)
		2411	Gehaktbal	Meatball	2364	Kipschnitzel gepaneerd	Breaded chicken schnitzel
		2550	Shoarmarol	Shawarma roll	2365	Cordon bleu (gewone)	Cordon blue (regular)
		2552	Kipkrokant schnitzel (gepaneerd met cornflakes)	Crispy chicken schnitzel (breaded with cornflakes)	2766	Worst rook-	Smoked sausage
		2555	Viandel	Viandel (Dutch meat snack, similar to frikandel)	2767	Worst rook-	Smoked sausage
		2654	Kip filet	Chicken fillet	2826	Schnitzel (vlees) gevulde	Stuffed schnitzel (meat)
		2766	Worst rook-	Smoked sausage	2899	Balkenbrij	Balkenbrij (Dutch meat-and-grain loaf)
		2767	Worst rook-	Smoked sausage	2907	Spek uitgebakken kant-en-klaar	Pre-cooked bacon (ready toe at)
		2826	Schnitzel (vlees) gevulde	Stuffed schnitzel (meat)	3003	Worst knak- runder blik/glas	Frankfurter sausages - canned/jarred
		2827	Schnitzel (vlees) gevulde	Stuffed schnitzel (meat)	3017	Kalf gehakt	Ground veal
		2836	Worst grill- met kaas	Grilled sausage with cheese	3018	Kalfsoester	Veal tenderloin
		2889	Kipnuggets	Chicken nuggets	3019	Kalf lap mager	Lean veal cut
		2899	Balkenbrij	Balkenbrij (Dutch meat-and-grain loaf)	3022	Kalf lap, n.s.	Veal cut, not specified
		2907	Spek uitgebakken kant-en-klaar	Pre-cooked bacon (ready toe at)	3023	Kalfsvleesreepjes	Veal strips
		2997	Worst grill-	Grilled sausage	3024	Varken nasi/bamivlees	Pork (for nasi/bami dishes)
		3001	Kalkoenfilet (vleeswaar)		3025	Spek reepjes/blokjes mager gerookt	Lean smoked bacon strips/cubes
		3017	Kalf gehakt	Ground veal	3027	Varken shoarma/gyrosvlees	Pork shawarma/gyros meat
		3019	Kalf lap mager	Lean veal cut	3030	Worst braad-	Pan-frying sausage
		3020	Kalf kotelet	Veal chop	3031	Varken sparerib	Pork spareribs
		3022	Kalf lap, n.s.	Veal cut, not specified	3032	Rund rib-eye	Beef rib-eye
		3024	Varken nasi/bamivlees	Pork (for nasi/bami dishes)	3033	Rund T-bone steak	Beef T-bone steak
		3025	Spek reepjes/blokjes mager gerookt	Lean smoked bacon strips/cubes	3039	Kalfsschnitzel ongepaneerd	Veal schnitzel
		3027	Varken shoarma/gyrosvlees	Pork shawarma/gyros meat	3139	Kip gehakt	Ground chicken (chicken mince)
		3028	Rund lap borst-	Beef brisket cut	5163	Gehaktbal	Meatball
		3030	Worst braad-	Pan-frying sausage	5475	Kipschnitzel krokant	Breaded chicken schnitzel
		3031	Varken sparerib	Pork spareribs			
		3032	Rund rib-eye	Beef rib-eye			
		3033	Rund T-bone steak	Beef T-bone steak			
		3139	Gehakt, n.s.	Ground meat, not specified			
		3223	Worst grill-	Grilled sausage			
		3229	Salade kip-sate	Chicken sate salade			
		5163	Gehaktbal	Meatball			
		5195	Vet kippen	Chicken fat			
		5475	Kipschnitzel krokant	Breaded chicken schnitzel			
Fish, shellfish and amphibians	Fish, shellfish and amphibians (warm meals)	111	Mosselen	Mussels	111	Mosselen	Mussels
		113	Haring	Herring	113	Haring	Herring
		114	Vis mager	Fish lean	114	Vis mager	Fish lean
		116	Vis vet	Fish fat	116	Vis vet	Fish fat
		347	Bokking gerookt	Herring smoked	347	Bokking gerookt	Herring smoked
		348	Garnalen Hollandse	Dutch Shrimp	348	Garnalen Hollandse	Dutch Shrimp
		350	Haring gezouten	Salted herring	350	Haring gezouten	Salted herring
		352	Kreeft gekookt	Lobster cooked	352	Kreeft gekookt	Lobster cooked
		353	Makreel	Mackerel	353	Makreel	Mackerel
		354	Oesters	Oysters	354	Oesters	Oysters
		355	Sardines in olie blik	Sardines is oil (canned)	355	Sardines in olie blik	Sardines is oil (canned)
		602	Zalm blik	Salmon (canned)	602	Zalm blik	Salmon (canned)
		603	Paling gerookt	Eel smoked	603	Paling gerookt	Eel smoked
		815	Vissticks	Fish Sticks	815	Vissticks	Fish Sticks
		818	Lekkerbek	Fried fish	818	Lekkerbek	Fried fish
		819	Kabeljauw	Cod	819	Kabeljauw	Cod
		918	Schol	Plaice	918	Schol	Plaice
		1096	Zalm gerookt	Salmon smoked	1096	Zalm gerookt	Salmon smoked
		1100	Haring in (zoet)zuur	Herring pickled	1100	Haring in (zoet)zuur	Herring pickled
		1584	Inktvis	Squid	1584	Inktvis	Squid
		1585	Heilbot gerookt	Halibut smoked	1585	Heilbot gerookt	Halibut smoked
		1586	Makreelfilet gerookt	Mackerel fillet smoked	1586	Makreelfilet gerookt	Mackerel fillet smoked
		1587	Zalm kweek	Farmed salmon	1587	Zalm kweek	Farmed salmon
		1588	Ansjovis in olie blik	Anchovies in oil (canned)	1588	Ansjovis in olie blik	Anchovies in oil (canned)
		1589	Tonijn in olie blik	Tuna in oil (canned)	1589	Tonijn in olie blik	Tuna in oil (canned)
		1590	Tonijn in water blik	Tuna in water (canned)	1590	Tonijn in water blik	Tuna in water (canned)
		1608	Makreel	Mackerel	1608	Makreel	Mackerel
		1609	Makreel in olie blik	Mackerel in oil (canned)	1609	Makreel in olie blik	Mackerel in oil (canned)
		1610	Zalm kweek	Farmed salmon	1610	Zalm kweek	Farmed salmon
		1614	Schelvis	Haddock	1614	Schelvis	Haddock
		1619	Tong	Sole	1619	Tong	Sole
		1622	Baars rood	Red perch	1622	Baars rood	Red perch
		1623	Zeewolf	Catfish / Wolffish	1623	Zeewolf	Catfish / Wolffish
		1631	Garnalen in water blik	Shrimp in water (canned)	1631	Garnalen in water blik	Shrimp in water (canned)
		1632	Inktvis	Squid	1632	Inktvis	Squid
		1899	Jacobsschelpen	Scallops	1899	Jacobsschelpen	Scallops
		2297	Tonijn	Tuna	2297	Tonijn	Tuna
		2474	Kibbeling	Battered fish chunks	2474	Kibbeling	Battered fish chunks
		2475	Zalmpate/mousse	Salmon pâté	2475	Zalmpate/mousse	Salmon pâté
		2765	Pangasius	Pangasius	2765	Pangasius	Pangasius
		3070	Tonijn	Tuna fat	3070	Tonijn	Tuna fat
		3071	Tilapia	Tilapia	3071	Tilapia	Tilapia
		3137	Bakkeljauw	Salt cod	3137	Bakkeljauw	Salt cod
		3205	Ansjovis	Anchovy	3205	Ansjovis	Anchovy
		3252	Krabsticks fantasie	Crabsticks	3252	Krabsticks fantasie	Crabsticks
		3256	Sardines gegrild	Grilled sardines	3256	Sardines gegrild	Grilled sardines
		3319	Koolvis (Alaska)	Alaska pollock	3319	Koolvis (Alaska)	Alaska pollock
		3320	Gamba	Prawns	3320	Gamba	Prawns
		5266	Zeevruchten bereid	Seafood	5266	Zeevruchten bereid	Seafood

Supplementary Table 4. Overview of intake recommendations per age group used in our model

		EAA (mg/kg/bodyweight) (WHO recommendations)									Vitamins (EFSA recommendations)											Minerals (EFSA recommendations)								
Age group	Total protein intake (g/kg/bodyweight)	Tryptophan	Threonine	Sulfur amino acids#	Aromatic amino acids##	Histidine	Lysine	Valine	Isoleucine	Leucine	Vitamin A (RAE)	Vitamin B1 (mg)	Vitamin B2 (mg)	Vitamin B3 (mg)	Vitamin B6 (mg)	Folate (µg)	Vitamin B12 (µg)	Vitamin C (mg)	Vitamin D (µg)**	Vitamin E (mg)	Vitamin K (µg)	Calcium (mg)	Cupper (mg)	Iron (mg)	Iodine (µg)***	Potassium (mg)	Manganese (mg)	Phosphorus (mg)	Selenium (µg)	Zink (mg)
Boys 1-3	1,01	6,4	23	22	40	15	45	36	27	54	300	0,3	0,5	4	0,4	85	0,7	25	10	4	30	500	0,3	8	70	1400	85	470	20	5
Girls 1-3	1,01	6,4	23	22	40	15	45	36	27	54	300	0,3	0,5	4	0,4	85	0,7	25	10	4	30	500	0,3	8	70	1400	85	470	20	5
Boys 4-8	0,89	4,8	18	18	30	12	35	29	23	44	400	0,5	0,7	7	0,7	150	1,3	40	10	6	55	700	0,5	9	120	2000	200	540	30	7
Girls 4-8	0,89	4,8	18	18	30	12	35	29	23	44	400	0,5	0,7	7	0,7	150	1,3	40	10	6	55	700	0,5	9	120	2000	200	540	30	7
Boys 9-13	0,9	4,8	18	17	30	12	35	29	22	44	600	0,8	1	11	1,1	225	2	50	10	8	60	1200	0,7	11	150	3300	280	700	40	11
Girls 9-13	0,91	4,8	18	17	30	12	35	29	22	44	600	0,8	1	11	1,1	225	2	50	10	7	60	1100	0,7	11	150	2900	280	700	40	8
Boys 14-18	0,88	4,5	17	16	28	12	33	28	21	45	900	1,1	1,5	17	1,5	300	2,8	75	10	10	75	1200	0,9	11	150	3500	350	700	60	12
Girls 14-18	0,85	4,5	17	16	28	12	33	28	21	45	700	1,1	1,1	13	1,5	300	2,8	75	10	8	75	1100	0,9	15	150	3100	280	700	50	9
Men 19-30	0,83	4	15	15	25	10	30	26	20	39	900	1,1	1,5	17	1,8	300	2,8	75	10	10	120	1000	0,9	9	150	3500	350	600	60	9
Women 19-30	0,83	4	15	15	25	10	30	26	20	39	700	1,1	1,1	13	1,5	300	2,8	75	10	8	90	1000	0,9	15	150	3100	280	600	50	7
Men 31-50	0,83	4	15	15	25	10	30	26	20	39	900	1,1	1,5	17	1,8	300	2,8	75	10	10	120	1000	0,9	9	150	3500	350	600	60	9
Women 31-50	0,83	4	15	15	25	10	30	26	20	39	700	1,1	1,1	13	1,5	300	2,8	75	10	8	90	1000	0,9	15	150	3100	280	600	50	7
Men 51-70	0,83	4	15	15	25	10	30	26	20	39	900	1,1	1,5	17	1,8	300	2,8	75	10	10	120	1100	0,9	9	150	3500	350	600	60	9
Women 51-70	0,83	4	15	15	25	10	30	26	20	39	700	1,1	1,1	13	1,5	300	2,8	75	10	8	90	1100	0,9	15	150	3100	280	600	50	7
Men 71-79	1,2*	4	15	15	25	10	30	26	20	39	900	1,1	1,5	17	1,8	300	2,8	75	20	10	120	1200	0,9	9	150	3500	350	600	60	9
Women 71-79	1,2*	4	15	15	25	10	30	26	20	39	700	1,1	1,1	13	1,5	300	2,8	75	20	8	90	1200	0,9	9	150	3100	280	600	50	7

*ESPEN recommendation

**Dutch Health Counsil recommendation

***WHO recommendation

#Recommendation of Methionine combined with Cysteine

##Recommendation of Phenylalanine combined with Tyrosine
